# Correction of leg-length discrepancy among meat cutters with low back pain: a randomized controlled trial

**DOI:** 10.1186/s12891-019-2478-3

**Published:** 2019-03-14

**Authors:** Satu Rannisto, Annaleena Okuloff, Jukka Uitti, Markus Paananen, Pasi-Heikki Rannisto, Antti Malmivaara, Jaro Karppinen

**Affiliations:** 1 Finnish Institute of Occupational Health, Oulu, Tampere, Finland; 2 Information Systems Unit, National Institute for Health and Welfare, Helsinki, Finland; 3 Clinic of Occupational Medicine, Tampere University Hospital, Tampere, Finland; 4 Faculty of Medicine and Life Sciences, University of Tampere, Arvo Ylpön katu 34, 33014 Tampere Yliopisto, Finland; 5 Medical Research Centre Oulu, University of Oulu and Oulu University Hospital, Oulu, Finland; 6 Faculty of Social Sciences, Health sciences, University of Tampere, Tampere, Finland; 7 Centre for Health and Social Economics, National Institute for Health and Welfare, Helsinki, Finland; 8 Orton Orthopaedic Hospital and Orton Research Institute, Orton Foundation, Helsinki, Finland; 9 Center for Life Course Health Research, University of Oulu, Oulu, Finland

**Keywords:** Leg-length discrepancy, Low back pain, Insoles, Randomized controlled trial

## Abstract

**Background:**

The etiology of non-specific low back pain (LBP) is complex and not well understood. LBP is common and causes a remarkable health burden worldwide. Leg-length discrepancy (LLD) is potentially a risk factor for development of LBP, although this relationship has been questioned. Yet only one randomized controlled study (RCT) has been performed. The objective of our study was to evaluate the effect of insoles with leg-length discrepancy (LLD) correction compared to insoles without LLD correction among meat cutters in a RCT-design.

**Methods:**

The study population consisted 387 meat cutters who were over 35 years old and had been working 10 years or more. The LLD measurement was done by a laser ultrasound technique. All workers with an LLD of at least 5 mm and an LBP intensity of at least 2 on a 10-cm Visual Analog Scale were eligible. The LLD of all the participants in the intervention group was corrected 70%, which means that if the LLD was for example 10 mm the correction was 7 mm. The insoles were used at work for eight hours per day. The control group had insoles without LLD correction. The primary outcome was between-group difference in LBP intensity. Secondary outcomes included sciatic pain intensity, disability (Roland Morris), RAND-36, the Oswestry Disability Index, physician visits and days on sick leave over the first year. We used a repeated measures regression analysis with adjustments for age, gender and BMI. The hurdle model was used for days on sick leave.

**Results:**

In all, 169 workers were invited and 114 (67%) responded. Of them, 42 were eligible and were randomized to the intervention (*n* = 20) or control group (*n* = 22). The workers in the intervention group had a higher improvement in LBP intensity (− 2.6; 95% confidence intervals **−** 3.7 **– −** 1.4), intensity of sciatic pain (− 2.3; − 3.4 – − 1.07) and RAND-36 physical functioning (9.6; 1.6–17.6) and a lesser likelihood of sick leaves (OR -3.7; − 7.2 - -0.2).

**Conclusions:**

Correction of LLD with insoles was an effective intervention among workers with LBP and a standing job.

**Trial registration:**

ISRCTN11898558. Registration date 11. Feb 2011. BioMed Central Ltd.

## Background

The etiology of non-specific low back pain (LBP) is complex and not well understood. LBP is common and causes a remarkable health burden worldwide [1]. Leg-length discrepancy (LLD) is potentially a risk factor for the development of LBP, although this relationship has been questioned [2]. Several studies have found LLD to be associated with LBP [3–5], but opposite results have also been reported [6–9]. The interventions on LLD correction are based mostly on observational designs [3–5, 10]. Only one randomized controlled study (RCT) has been published so far, which showed promising results in favor of LLD correction [11].

We have previously established a new LLD measurement with a laser ultrasound range meter [12]. The measurement method showed an almost perfect agreement between repeated measurements with minimal systematic errors. Furthermore, the laser ultrasound technique was quick and easy to perform. Using this technique, we observed that LLD was associated with intensity of LBP and self-reported days with LBP during the past year among meat cutters engaged in standing work but not among customer service workers engaged in sedentary work [13]. We hypothesize that correction of LLD using shoe lifts (insoles) may reduce LBP, especially in standing jobs. Therefore, this study aimed to assess the efficacy of shoe lift intervention in a randomized controlled design among workers with a standing job.

## Methods

### The study population

The study population consisted of workers in the food industry (Atria Finland Ltd., Nurmo, Finland). The entire population of the pork cutting department was 387 meat cutters with a standing job. All workers who had been working in their jobs for at least 10 years and were at least 35 years old were invited to the LLD measurement. For the intervention, all meat cutters who agreed to participate and presented with an LLD of at least 5 mm and an LBP intensity of at least 2 on a 10-cm Visual Analog Scale (VAS) were recruited. We chose LLD of 5 mm or more because approximately 50% of the population meets this criterium [14] and we observed a similar finding in this study population with 48% have LLD of at least 5 mm [13]. The study was approved by the Ethics Committee of the Central Hospital of Southern Ostrobothnia (11/2006), and followed the principles of the Declaration of Helsinki. All participants took part on a voluntary basis and signed their informed consent.

### Measurement of LLD

In the assessment of inter-rater reliability, two physiotherapists from Seinäjoki University of Applied Sciences measured 20 healthy voluntary students with no known previous LLD (90% women, mean age 23 years (range 19–35 years) on two consecutive days, blinded to each other’s assessment. For the evaluation of intra-rater reliability, the same two physiotherapists measured the same 20 voluntary students in mixed order on two consecutive days [12]. The same two physiotherapists measured also the participants of this study but did not participate in the study otherwise.

LLD was measured by a laser ultrasound technique. The scanning head of the ultrasound apparatus was placed perpendicular to the tissue interface in the hip area. The distance to the floor was measured by the laser measure at the point of the highest rim of the femoral head. This non-invasive laser ultrasound technique has been described previously [12].

### Randomization

A statistician not involved in the study generated the randomization list via computer in blocks of 10 containing five active and five control intervention allocations listed in random order. An occupational physiotherapist assigned the patients a randomization number in the order that they telephoned her. Participants and the principal investigator were blinded to treatment allocation.

### Insoles for intervention

Intervention group workers were given insoles for both legs and a raised LLD-corrected insole for the shorter leg. The insoles (JalasFX2 insoles, size 35–50 ESD insoles) had elevation under the heel and were made by an experienced physiotherapist who did not participate in the study otherwise. The LLD of all the participants in the intervention group was corrected. The correction of LLD was 70%. Therefore, e.g. a 10-mm LLD was corrected by 7 mm with a remaining LLD of 3 mm. Participants in the intervention group used the LLD-corrected insoles at work for eight hours per day. Participants in the control group were given new insoles without LLD correction. All patients received the insoles from the same occupational physiotherapist who assigned the participants to the allocation of the randomization code but did not participate in the study otherwise.

### Outcomes

The primary outcome was the intensity of LBP during the past week using a 10-cm VAS. The secondary outcomes included health-related quality of life (RAND-36) [15], back-related disability (Roland Morris Disability Questionnaire, RMDQ) [16], the Oswestry Disability Index (ODI) [17], and intensity of sciatic pain during the past week (measured on the 10-cm VAS). These outcomes were assessed at baseline, three months, six months and one year. Additional secondary outcomes included number of days on sick leave due to LBP and visits to a physician for all reasons during the preceding year. Days on sick leave due to LBP (diagnoses M50–54 in ICD-10) were collected comprehensively from the occupational health care registers of Atria Finland Ltd. from the preceding year before the intervention and from the baseline to the one-year follow up.

### Statistical methods

The SAS v. 9.4 program was used for the analyses. The GLIMMIX procedure was used for all analyses except the hurdle regression analysis, which was analyzed using the NLMIXED procedure. The data was analyzed using a repeated measures regression with time as a classification variable to prevent the model regressing the outcomes at different time points to the mean of the groups. The intensities of low back and sciatic pain were normally distributed. Poisson and negative binomial distributions were used for the count measures (RAND-36 summary scores, RMDQ results and number of visits to a physician). The number of days on sick leave had an excess of zeros compared to the Poisson and even negative binomial distributions. Therefore, the hurdle model with a negative binomial count part and a binomial probability part was used. In the analyses, the independent variables were intervention group, time, time*group interaction, age, gender and BMI. The treatment difference is the estimate of the group*time interaction in the case of normal distribution. In the generalized linear models (here in the cases of Poisson, negative binomial and binomial distributions), the interaction estimate is calculated on the model (link function) scale. The data scale treatment differences were calculated using the LSMESTIMATE statement in GLIMMIX and the ESTIMATE statement in NLMIXED.

## Results

The entire population of the pork cutting department was 387 meat cutters. In all, 169 pork meat cutters (31 females and 138 males) were eligible and 114 (67.5%) responded to the questions (26 females and 88 males). Of them, 72 were excluded (no LLD or no LBP; Fig. 1) while 42 (7 females and 35 males) had an LLD of at least 5 mm and an LBP intensity of at least 2 on a 10-cm VAS and agreed to participate. The workers were randomized into the intervention (*n* = 20) and the control groups (*n* = 22; Fig. 1).

**Fig. 1:**
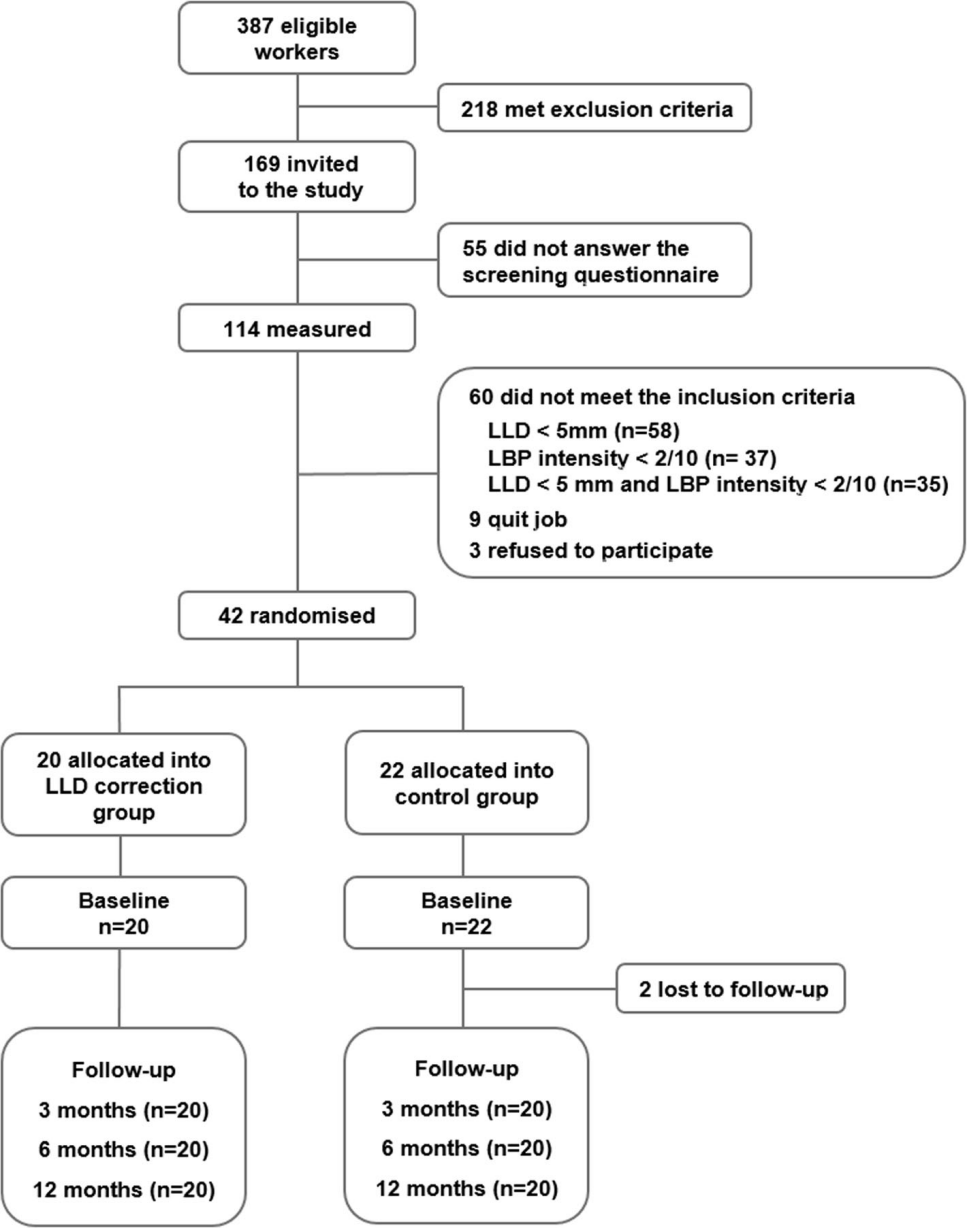
Flow chart of the study

The characteristics of the participants and non-participants are described in Table 1. The mean LLD was 9 mm in the study group, 7.9 mm in the control group and 3.5 mm among non-participants. The non-participants were more often females (33%) than the participants (intervention group 25% and control group 9%). The other characteristics did not differ between participants and non-participants. The differences between the intervention and control groups at the baseline were not statistically significant.

**Table 1:** Characteristics of the participants (*n* = 40) and non-participants (*n* = 72) at baseline

	Participants		
Variable	Intervention (*N* = 20)	Control (*N* = 20)	Non-participants (*n* = 72)
Age, mean (SD)	45.5 (9.3)	45.7 (7.6)	47.5 (7.5)
Gender, n (%), women	5 (25)	2 (10)	24 (33)
BMI, mean (SD)	26.6 (4.2)	26.8 (3.6)	27.5 (4.9)
Leg length discrepancy, mean (SD)	9.0 (2.8)	8.0 (3.4)	3.5 (3.7)
Number of days on sick leave due to LBP during the past year, mean (SD)	10.1 (10.2)	7.7 (16.3)	8.3 (18.6)
Visits to physician during the past year, mean (SD)	4.7 (2.0)	4.2 (2.4)	N/A
Current smoker, n (%)	4 (20)	6 (30)	18 (25)

Participant adherence to our study was good. All but one of the intervention group participants reported that they had used the elevated insoles during work hours. The scores of the primary outcome — intensity of LBP during the past week — decreased in both groups during the follow-up (Table 2, Fig. 2). The treatment difference (− 2.6; 95% confidence interval (CI) **-**3.7 **– -**1.4) favored the intervention group significantly (Table 2).

**Table 2:** Low back symptoms and disability scores at baseline, 3 months, 6 months and 12 months according to treatment group, within group differences and between group comparisons of difference from baseline to 12 months

Outcome measure	Mean (SD) crude scores		Difference between baseline and 12 months (95% CI) repeated measures regression^1^		Treatment difference (95% CI) repeated measures regression^1^
	Intervention	Control	Intervention	Control	
Intensity of LBP during last week					
Baseline	4.9 (1.5)	4.0 (1.5)			
3 months	3.8 (1.9)	3.4 (1.7)			
6 months	2.0 (1.1)	3.0 (1.9)			
12 months	1.6 (0.9)	3.1 (2.1)	**− 3.3 (− 4.3 to − 2.3)**	**−0.8 (− 1.9 to 0.4)**	**−2.6 (− 3.7 to − 1.4)**
Intensity of sciatica during last week					
Baseline	3.2 (1.9)	2.0 (2.0)			
3 months	2.6 (2.1)	1.7 (2.0)			
6 months	1.3 (1.0)	1.7 (1.8)			
12 months	0.8 (1.0)	1.9 (2.0)	**−2.3 (− 4.4 to − 1.3)**	**−0.1 (− 1.4 to 1.2)**	**− 2.3 (− 3.4 to − 1.1)**
Disability (Roland-Morris Disability Questionnaire)					
Baseline	5.8 (3.2)	5.0 (4.0)			
3 months	4.0 (3.6)	3.3 (1.9)			
6 months	3.6 (3.6)	3.7 (2.8)			
12 months	3.4 (3.3)	3.2 (2.3)	**−1.9 (−3.1 to − 0.7)**	**− 1.4 (−2.5. to − 0.3)**	−0.5 (− 1.3 to 0.3)
Oswestry Disability Index (NB)					
Baseline	18.2 (9.0)	12.2 (11.4)			
3 months	14.5 (7.3)	13.3 (9.1)			
6 months	13.2 (7.7)	11.8 (8.8)			
12 months	13.3 (9.3)	11.0 (9.8)	**−4.8 (−8.1 to − 1.5)**	−1.2 (−3.6 to 1.2)	−3.5 (−7.6 to 0.5)
Days on sick leave due to LBP during the past year					
Baseline	10.1 (10.2)	7.0 (15.7)	**−7.0 (−11.3 to − 2.6)** ^**2**^	− 5.0 (− 11.5 to 1.5)^2^	1.9 (− 5.5 to 9.3)^2^
12 months	2.4 (4.1)	6.2 (10.3)			
			**−52.9 (− 8.6 to − 97.2)** ^**3**^	27.4 (− 15.7 to 70.4)^3^	**− 80.3 (− 146.1 to − 14.5)** ^**3**^

*LLD* leg length discrepancy, *LBP* low back pain, *NB* negative binomial model, *95% CI* 95% confidence interval. Bold denotes significance

^1^Adjusted by age, BMI, gender and smoking. Hurdle model produces two kinds of estimates for difference between the baseline and 12 months and for the treatment difference:

^2^is the count part, the number of days on sick leave due to LBP and

^3^is the probability part for the likelihood of the sickness absence due to LBP

**Fig. 2:**
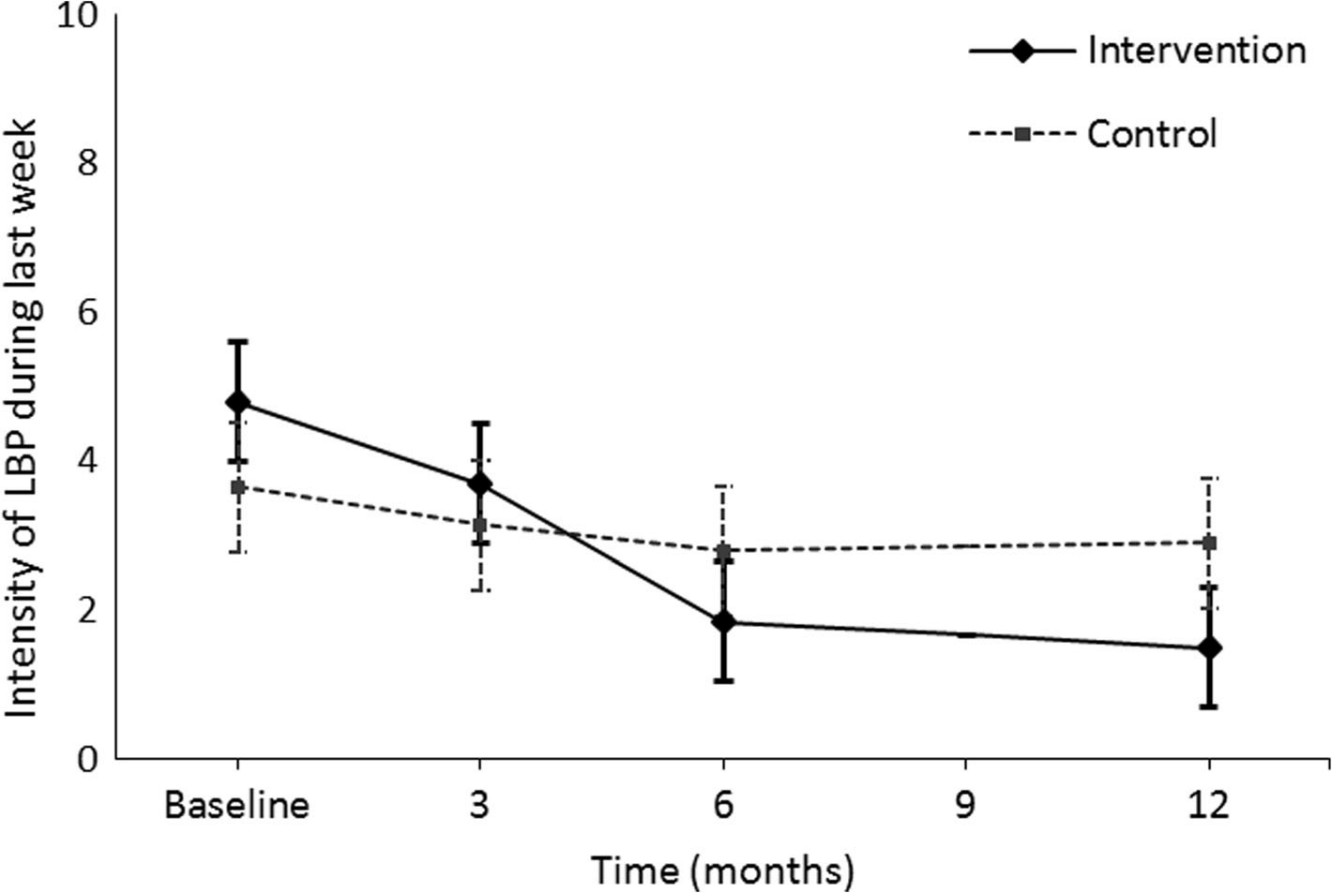
Mean intensity of LBP during past week and its 95% confidence intervals (CI) at each time point and in both intervention groups

Among the secondary outcomes, the intervention group improved in all measured outcomes while the control group changes appeared more random, ranging from improvement to no change/ symptoms getting worse (Table 2 and Table 3). The significant differences between the groups were in intensity of sciatica pain during the past week, RAND-36 physical functioning score and RAND-36 general health score. The sciatica pain decreased in the intervention group but remained the same in the control group (treatment difference − 2.3; 95% CI -3.4 – -1.1), while RAND-36 physical functioning scores increased in the intervention group but remained the same in the control group (treatment difference 9.6; 95% CI 1.6–17.6). Furthermore, RAND-36 general health scores increased in the intervention group and worsened in the control group (treatment difference 15.2; 95% CI 6.1–24.2). No significant differences were observed in the other RAND-36 scores or in the RMDQ results (Table 2 and Table 3).

**Table 3:** Health-related quality of life (RAND-36) outcomes at baseline, 3 months, 6 months and 12 months according to treatment group, within group differences and between group comparisons of difference from baseline to 12 months

Outcome measure	Mean (SD) crude scores		Difference between baseline and 12 months (95% CI) repeated measures regression^1^		Treatment difference (95% CI) repeated measures regression^1^
	Intervention	Control	Intervention	Control	
Physical functioning					
Baseline	78.8 (13.6)	82.0 (12.8)			
3 months	85.8 (10.3)	83.0 (13.6)			
6 months	88.0 (8.8)	82.8 (10.3)			
12 months	89.3 (8.0)	83.0 (13.2)	**10.5 (4.8 to 16.2)**	0.9 (−4.7 to 6.6)	**9.6 (1.6 to 17.6)**
Role-physical (NB)					
Baseline	60.0 (40.9)	66.3 (34.7)			
3 months	72.5 (26.8)	67.5 (35.4)			
6 months	78.8 (29.6)	70.0 (29.9)			
12 months	91.3 (18.6)	68.8 (37.1)	32.7 (−11.3 to 76.8)	1.3 (−38.0 to 40.5)	31.5 (−27.5 to 90.5)
Role-emotional (NB)					
Baseline	81.7 (35.0)	90.0 (26.7)			
3 months	93.3 (13.7)	88.3 (27.1)			
6 months	93.3 (23.2)	90.0 (21.9)			
12 months	96.7 (10.3)	91.7 (18.3)	14.9 (−8.6 to 38.5)	1.7 (−22.3 to 25.8)	13.2 (−20.4 to 46.9)
Energy/fatigue					
Baseline	65.0 (16.9)	73.5 (18.8)			
3 months	66.8 (17.0)	73.3 (18.7)			
6 months	75.5 (14.4)	69.3 (14.5)			
12 months	72.3 (11.8)	75.5 (13.2)	**7.1 (2.0 to 12.2)**	2.0 (−3.3 to 7.3)	5.1 (−2.2 to 12.5)
Mental health					
Baseline	74.8 (17.8)	81.4 (15.0)			
3 months	75.6 (16.9)	77.6 (16.9)			
6 months	78.8 (14.4)	79.8 (11.8)			
12 months	79.0 (12.3)	79.6 (13.2)	4.2 (−1.3 to 9.6)	−1.8 (−7.4 to 3.8)	6.0 (−1.8 to 13.7)
Social functioning					
Baseline	81.3 (18.4)	74.4 (16.5)			
3 months	83.1 (18.7)	80.0 (14.2)			
6 months	84.4 (19.8)	76.3 (9.0)			
12 months	92.5 (13.7)	85.0 (13.8)	**11.1 (5.3 to 16.8)**	**10.7 (5.1 to 16.2)**	0.4 (−7.6 to 8.4)
Bodily pain (NB)					
Baseline	54.6 (17.2)	53.9 (22.4)			
3 months	59.8 (15.1)	58.6 (20.3)			
6 months	63.0 (12.8)	59.6 (23.1)			
12 months	73.5 (17.1)	60.3 (21.0)	**18.7 (4.7 to 32.7)**	6.5 (−5.9 to 19.0)	12.2 (−6.6 to 30.1)
General health (NB)					
Baseline	55.5 (16.0)	64.0 (24.5)			
3 months	61.8 (15.7)	60.5 (21.3)			
6 months	61.8 (15.1)	61.5 (22.2)			
12 months	63.8 (17.0)	57.3 (24.0)	**8.6 (2.2 to 15.1)**	**−6.5 (−11.3 to − 1.7)**	**15.2 (6.1 to 24.2)**

*LLD* leg length discrepancy, *LBP* low back pain, *NB* negative binomial model, *95% CI* 95% confidence interval. Bold denotes significance

^1^Adjusted by age, BMI, gender and smoking

From the hurdle model, the number of days on sick leave due to LBP decreased in both groups and the difference between the groups was not significant (Table 2 and Fig. 3). However, the probability of sick leave absence due to LBP decreased in the intervention group and increased in the control group (Fig. 4). The treatment difference for the probability was − 3.7 (95% CI -7.2 – -0.2) on the model scale and − 80.3% (95% CI -146.1 – -14.5) on the data scale (Table 2). There was no difference in the number of visits to the physician between the groups.

**Fig. 3:**
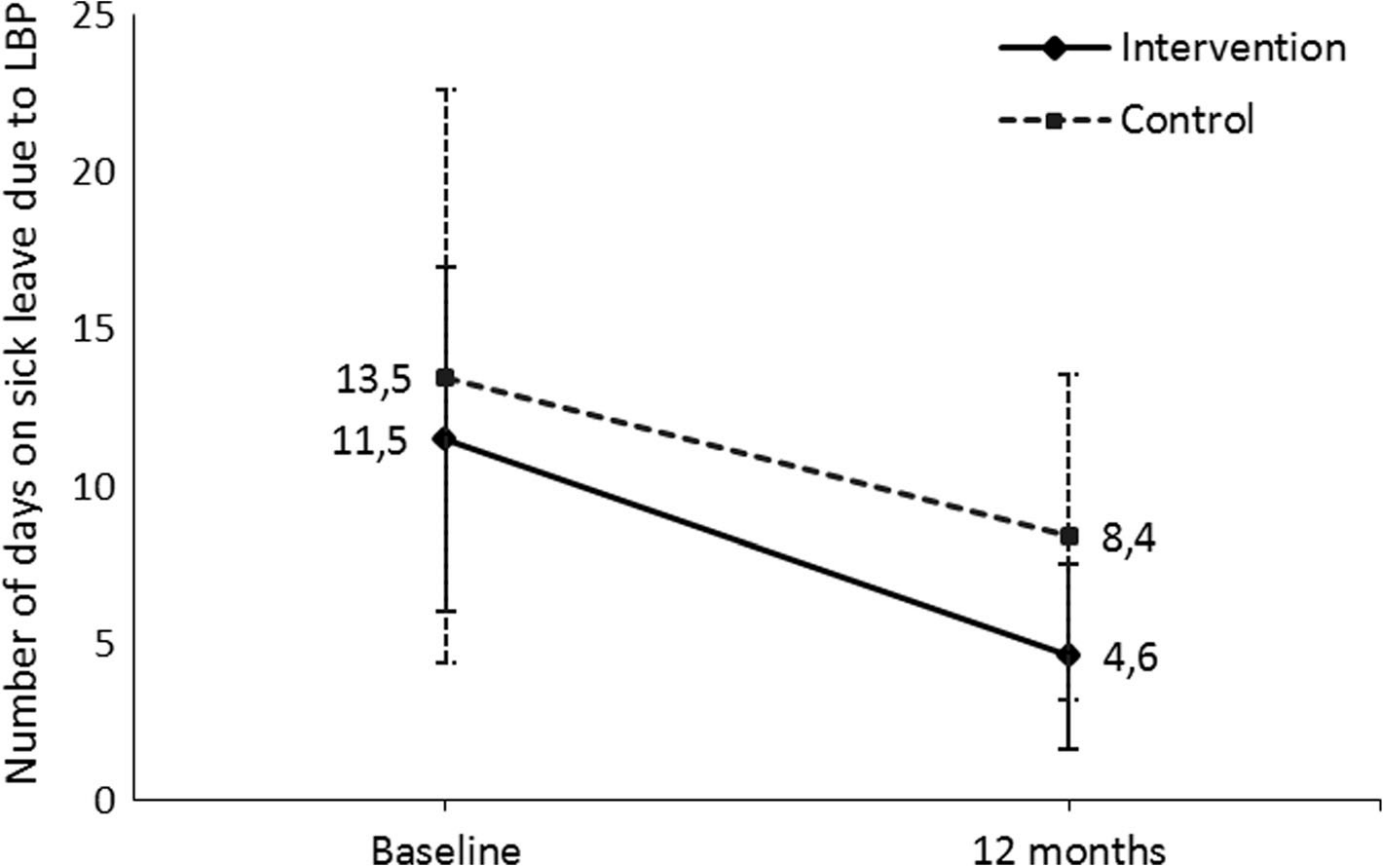
Probability of sickness absence due to LBP during the past year and its 95% confidence intervals (CI) at each time point and in both intervention groups

**Fig. 4:**
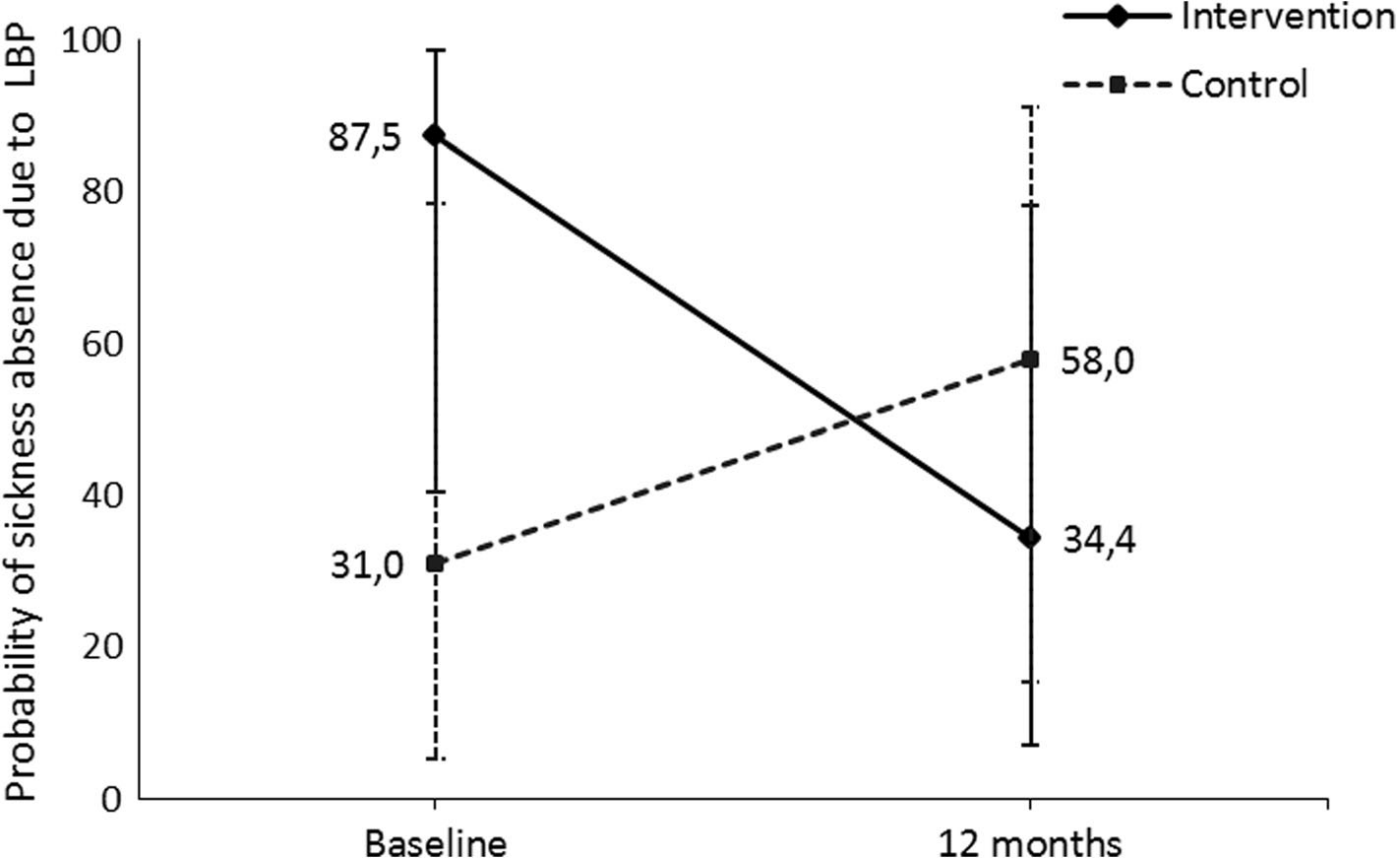
Mean number of days on sick leave due to LBP during the past year and its 95% confidence intervals (CI) at each time point and in both intervention groups

## Discussion

There are a few controlled studies that explore the association between LLD and LBP. Among meat cutters in our study, LLD was corrected by 70% using insoles with LLD correction compared to insoles without LLD correction in a randomized design. Correction of LLD resulted in significant improvements to intensity of LBP, intensity of sciatic pain and RAND-36 physical functioning and decreased the likelihood of sick leaves.

Most of the few studies incorporating the use of insoles in LLD show positive effects among LBP patients but are mostly based on a small number of participants. In the only RCT published so far [11], the investigators measured 33 patients using ultrasounds. The patients, who had an LLD of 10 mm or less, were randomized into two groups. In 22 patients, LLD was corrected by applying individually fitted shoe inserts. In 11 patients, LLD was not corrected. The researchers started with a 2-mm elevation, and further 2 mm elevations were added every second day until the desirable height was achieved. The LLD correction was equal to the original LLD minus 10%. During the follow-up time of 12 weeks, five patients had complete pain relief and 16 had substantial pain reduction, ranging between 33 and 72%. These results are in line with our results.

The results of other intervention studies are also in favor of LLD correction. However, the study designs and methodologies vary. Friberg [3] measured 789 patients with LBP in his study as well as 359 symptom-free patients using a radiographic method, and a statistically highly significant correlation of the symptoms and LLD were observed. Of those patients, 320 with LLD and LBP were given shoe lifts and 96 were symptom-free in follow-up at six months. In observational studies, Gofton [5] performed a retrospective study of 10 patients who had suffered with LBP for years. LLD was 10 mm or more and mostly measured in a clinical way. The follow-up was from 3 to 11 years and by questioning whether pain relief was major or complete. Golightly and colleagues [10] had 12 patients who were measured by radiography. The majority of those patients had a clinically significant decrease in their general pain symptoms and pain associated with standing.

Giles and Taylor [4] evaluated 1309 patients with LBP, and 244 had LLD greater than 10 mm (measured by radiography). The two groups were randomly selected. The heel raise was equal to the difference in leg length. In the first group, 35 patients were given both shoe-raise therapy and lumbo-sacral manipulation. Only shoe-raise therapy was given to 15 patients, but if LBP persisted for one month, those patients were also manipulated. The patients were asked to measure their LBP on a 0–4 scale, where 0 is none and 4 is unbearable. In the shoe-therapy group, the score was 2.4 at the beginning but lowered to 1.1 after one or two years. In the shoe-therapy and lumbo-sacral manipulation group, the score was almost 5 at the beginning but lowered to 1.3 after one or two years. The results are not comparable with our study because they used a mixed methods approach.

Criteria for LLD correction are not clearly defined in previous studies, and they mostly corrected for an LLD of 10 mm or more. Previous lift studies have varied in the total amount of lift correction used in the context of treating patients with LLD and LBP. Giles and Taylor [4] utilized shoe or heel lifts equal to the magnitude of the LLD as determined by radiograph. Friberg [3] used lifts that were a few millimeters less than the LLD measured by radiograph. Defrin [11] used lifts equal to the LLD minus 10%, and LLD was corrected step by step. Also, Golightly [10] corrected LLD step by step based on pain relief. There are thus no valid criteria for relevant LLD correction.

We decided to correct for an LLD of 5 mm or more. In our study, each participants LLD was corrected by 70% using heel lifts, and all corrections were made simultaneously because we had no opportunity for a step-by-step method. No other treatments were given. Thus, our study was an intervention that yielded LLD correction without confusing treatments. Also, our LLD criteria and the criteria for its correction proved to be relevant based on the positive results of the intervention. The correction of a smaller measure of LLD than in earlier studies seems to be beneficial, even among this small population of employees with standing work.

The strengths of this study are the use of reliable methodology, from the measurement of LLD to the outcome variables, and the strong commitment of the study population to the intervention. As outcomes we used measurement instruments, which are validated in nonspecific LBP [18]. However, a recent systematic review questioned the construct validity of health-related quality of life instruments [19]. The between-group treatment difference from baseline to one year in pain intensity (both low back and sciatic pain) exceeded the values defined for minimally clinically important change [20, 21]. Moreover, all sick leave information and the right diagnoses were recorded precisely in the Atria occupational health systems. The study had a small population, however, and we were not able to differentiate why patients visited their physicians.

The studies of the relationship between LLD and LBP seem to be of poor quality and consist of an insufficient number of participants producing contradictory results. Our study showed clinically important improvements in patients’ symptoms and workability after correction of LLD. However, we recommend more randomized controlled studies among different working populations with proper study designs and different criteria for LLD correction.

## Conclusion

LLD of 5 mm or more, measured with a reliable measurement method, seems to be worth correcting using shoe lifts among persons having a standing job. The correction is possible to be done also in the context of occupational health services. The correction of LLD in our study demonstrably reduced subjective pain and the probability of taking sick leave days.

## Abbreviations

BMI: Body mass index
LBP: Low back pain
LLD: Leg length discrepancy
NB: Negative binomial model
ODI: Oswestry Disability Index
RCT: Randomized controlled trial
RMDQ: Roland Morris Disability Questionnaire
VAS: Visual analog scale
